# Shift in diagnostic classification of migraine after initiation of preventive treatment with eptinezumab: post hoc analysis of the PROMISE studies

**DOI:** 10.1186/s12883-022-02914-9

**Published:** 2022-10-25

**Authors:** Patricia Pozo-Rosich, David W. Dodick, Anders Ettrup, Joe Hirman, Roger Cady

**Affiliations:** 1 Neurology Department, Headache Unit, Vall d’Hebron, University Hospital and Research Institute, Universitat Autonoma de Barcelona, Barcelona, Spain; 2 Headache and Neurological Pain Research Group, Department de Medicina, Vall d’Hebron, University Hospital and Research Institute, Universitat Autonoma de Barcelona, Barcelona, Spain; 3 Mayo Clinic, Scottsdale, AZ USA; 4 Atria Institute, New York, NY USA; 5 H. Lundbeck A/S, Copenhagen, Denmark; 6 Pacific Northwest Statistical Consulting, Inc., Woodinville, WA USA; 7 Lundbeck LLC, Deerfield, IL USA; 8 RK Consults, Ozark, MO USA; 9 Missouri State University, Springfield, MO USA

**Keywords:** Migraine, Prevention, Efficacy, Eptinezumab

## Abstract

**Background:**

Monthly headache frequency directly correlates with personal/societal burden and impacts severity and preventive treatment decisions. This post hoc analysis identified shifts from higher to lower frequency headache categories over 6 months in patients with migraine participating in the PROMISE clinical trials receiving two eptinezumab doses.

**Methods:**

Headache frequency at baseline and over study months 1–6 was categorized into 4 groups: chronic migraine (CM; ≥ 15 monthly headache days [MHDs]), high-frequency episodic migraine (HFEM; 10–14 MHDs), low-frequency episodic migraine (LFEM; 4–9 MHDs), and ≤ 3 MHDs. Outcomes included the percentage of patients within each MHD category, the percentage of patients improving by ≥ 1 MHD category, and the number of months with reduction of ≥ 1 MHD category. Data from patients who received approved eptinezumab doses (100 mg or 300 mg) or placebo were included.

**Results:**

Mean headache frequency at baseline in PROMISE-1 was 10 MHDs; most patients were classified as having HFEM (48.6%) or LFEM (43.9%). At Month 1, 62/221 (28.1%), 75/222 (33.8%), and 45/222 (20.3%) patients who received eptinezumab 100 mg, 300 mg, and placebo had ≤ 3 MHDs, with 97/221 (43.9%), 108/222 (48.6%), and 84/222 (37.8%), respectively, falling below the diagnostic EM threshold at Month 6. More than one-third (79/221 [35.7%], 83/222 [37.4%], and 68/222 [30.6%] of patients in the eptinezumab 100 mg, 300 mg, and placebo groups, respectively), had 6 months of reduction of ≥ 1 frequency category. At baseline in PROMISE-2, mean headache frequency was 20.5 MHDs. All patients (100%) in the eptinezumab 100 mg and placebo groups had CM, as did 99.4% of patients receiving eptinezumab 300 mg. At Month 1, 209/356 (58.7%), 216/350 (61.7%), and 167/366 (45.6%) patients treated with eptinezumab 100 mg, 300 mg, and placebo had ≤ 14 MHDs, with 240/356 (67.4%), 249/350 (71.1%), and 221/366 (60.4%), respectively, falling below CM threshold at Month 6. Additionally, 153/356 (43.0%), 169/350 (48.3%), and 116/366 (31.7%) patients in the eptinezumab 100 mg, 300 mg, and placebo groups, respectively, had 6 months of reduction of ≥ 1 frequency category.

**Conclusion:**

In the PROMISE studies, episodic and chronic migraine patients treated with eptinezumab were more likely to reduce their headache frequency versus placebo, which directly and in a sustained way improved their diagnostic category classification.

**Trial registration:**

ClinicalTrials.gov Identifier: NCT02559895, NCT02974153.

## Introduction

Headache frequency varies widely across the migraine spectrum and, in individual patients, may increase or decrease as their migraine worsens or improves [1, 2]. Because the individual and societal impacts of migraine increase with monthly headache day frequency [3–8], it is important to quantify changes in frequency occurring with preventive treatment. Furthermore, access to preventive treatment remains largely driven by the number of headache days and diagnostic migraine classification (e.g., episodic migraine [EM] or chronic migraine [CM]), despite calls to consider factors other than headache frequency when determining the need for preventive intervention [9–12].

Eptinezumab (Vyepti™, Lundbeck Seattle BioPharmaceuticals, Inc., Bothell, WA, USA) is a calcitonin gene-related peptide antagonist approved for migraine prevention in adults [13–15]. The preventive efficacy and safety of eptinezumab 100 mg and 300 mg administered intravenously every 12 weeks have been demonstrated across the spectrum of 4–26 migraine days per month [16–24]. In the phase 3 randomized, double-blind, placebo-controlled PROMISE studies, eptinezumab administered every 12 weeks significantly reduced migraine frequency, with onset of preventive efficacy demonstrated on Day 1 after dosing [17, 18]. In patients with EM (PROMISE-1), eptinezumab 100 mg and 300 mg reduced monthly migraine days (MMDs) over Weeks 1–12 by 3.9 (*P* = 0.0182 vs placebo) and 4.3 (*P* = 0.0001 vs placebo) days, respectively [18]. In patients with CM (PROMISE-2), eptinezumab 100 mg and 300 mg reduced MMDs over Weeks 1–12 by 7.7 (*P* < 0.0001 vs placebo) and 8.2 (*P* < 0.0001 vs placebo) days, respectively, and reduced monthly headache days (MHDs) over the same time period (–8.2 and –8.8 days, respectively; differences vs placebo [95% CI], –1.7 [–2.6, –0.9] and –2.3 [–3.2, –1.4], respectively) [17]. The objective of this post hoc analysis of data from the PROMISE studies was to identify the proportions of patients with migraine shifting from higher-frequency classification headache categories to lower-frequency headache categories over the first 6 months of treatment.

## Methods

Data were from the randomized, double-blind, placebo-controlled PROMISE studies [17, 18]. PROMISE-1 (NCT02559895) evaluated the safety and efficacy of eptinezumab 30 mg, 100 mg, and 300 mg in adults (18‒75 years of age) with a greater than 12-month history of EM, defined as ≤ 14 headache days per month, with ≥ 4 migraine days per month in the 3 months prior to screening [18]. Only data from patients who received approved doses (100 mg or 300 mg) were included in the current analysis. PROMISE-2 (NCT02974153) evaluated the safety and efficacy of eptinezumab 100 mg or 300 mg in adults (18‒65 years of age, inclusive) with a greater than 12-month history of CM, defined as ≥ 15 to ≤ 26 headache days and ≥ 8 migraine days during the 28-day screening period [17]. In both studies, eptinezumab was administered intravenously once every 12 weeks [17, 18].

Headache frequency at baseline and over study Months 1–6 was categorized into four groups for migraine category: CM (≥ 15 MHDs), high-frequency episodic migraine (HFEM; 10–14 MHDs), low-frequency episodic migraine (LFEM; 4–9 MHDs), and ≤ 3 MHDs [6, 8, 25].

Outcomes included the percentage of patients within each MHD category, the percentage of patients improving by ≥ 1 MHD category, and the sustained response with reduction of ≥ 1 MHD category. Data from patients who received approved eptinezumab doses (100 mg or 300 mg) or placebo were included. For PROMISE-2, outcomes in the subgroup of patients with medication-overuse headache (MOH) were also examined.

## Results

### Patients

A total of 443 adults received eptinezumab 100 mg or 300 mg in PROMISE-1 (100 mg, *n* = 221; 300 mg, *n* = 222) and 222 received placebo [18]. In PROMISE-2, 706 adults received eptinezumab (100 mg, *n* = 356; 300 mg, *n* = 350) and 366 received placebo [17]. Selected baseline demographic and clinical characteristics are summarized in Table 1; additional characteristics have been previously reported [17, 18].

**Table 1:** Select baseline demographic and clinical characteristics

	**PROMISE-1**			**PROMISE-2**		
	Eptinezumab 100 mg *n* = 223	Eptinezumab 300 mg *n* = 224	Placebo *n* = 222	Eptinezumab 100 mg *n* = 356	Eptinezumab 300 mg *n* = 350	Placebo *n* = 366
Mean (SD) age, years	40.0 (10.7)	40.2 (11.7)	39.9 (11.7)	41.0 (11.7)	41.0 (10.4)	39.6 (11.3)
Sex, *n* (%) female	179 (80.3)	199 (88.8)	186 (83.8)	307 (86.2)	314 (89.7)	325 (88.8)
Mean (SD) MHDs	10.0 (3.0)	10.1 (3.1)	9.9 (2.8)	20.4 (3.1)	20.4 (3.2)	20.6 (3.0)
Mean (SD) MMDs	8.7 (2.9)	8.6 (2.9)	8.4 (2.7)	16.1 (4.6)	16.1 (4.8)	16.2 (4.6)
MOH diagnosis, *n* (%)	‒	‒	‒	139 (39.0)	147 (42.0)	145 (39.6)
Diagnostic category, *n* (%)						
CM (≥ 15 MHDs)	16 (7.2)	20 (9.0)	14 (6.3)	356 (100)	348 (99.4)	366 (100)
HFEM (10–14 MHDs)	102 (46.2)	107 (48.2)	114 (51.4)	0 (0)	2 (0.6)	0﻿ (0)
LFEM (4–9 MHDs)	103 (46.6)	95 (42.8)	94 (42.3)	0﻿ (0)	0﻿ (0)	0﻿ (0)
≤ 3 MHDs	0 (0)	0 (0)	0 (0)	0﻿ (0)	0﻿ (0)	0﻿ (0)

*CM* Chronic migraine, *HFEM* High-frequency episodic migraine, *LFEM* Low-frequency episodic migraine, *MHD* Monthly headache days, *MMD* Monthly migraine days, *MOH* Medication-overuse headache, *SD* Standard deviation

Mean headache frequency at baseline in PROMISE-1 was 10 headache days per month, where 8.6 were migraine days [18]. For the purpose of this analysis, most patients in PROMISE-1 were classified as having HFEM or LFEM (eptinezumab 100 mg, 46.2% HFEM and 46.6% LFEM; eptinezumab 300 mg, 48.2% HFEM and 42.8% LFEM; placebo, 51.4% HFEM and 42.3% LFEM). A small number of patients in PROMISE-1 were classified as having CM at baseline; this is because the classification system for the current analysis was not completely consistent with how diagnoses were captured during the 28-day screening period.

At baseline in PROMISE-2, mean headache frequency was 20.5 MHDs, where 16.1 were migraine days [17]. All patients (100%) in the eptinezumab 100-mg and placebo groups had CM, as did 99.4% of patients receiving eptinezumab 300 mg. A total of 431/1072 (40.2%) patients in PROMISE-2 had an MOH diagnosis at baseline [17].

### Changes in diagnostic category in PROMISE-1

**Fig. 1:**
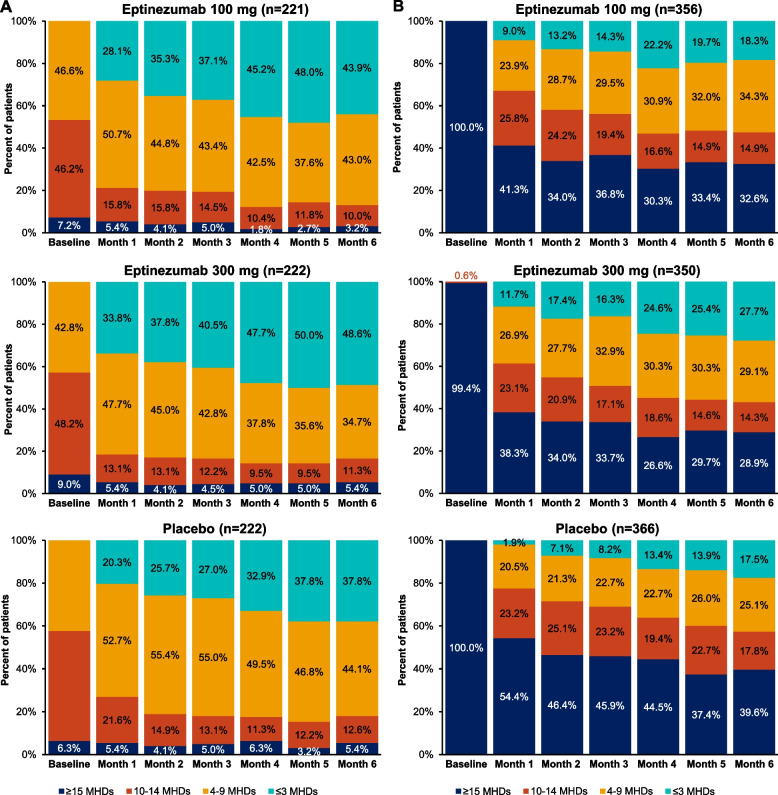
Percentage of patients in each diagnostic category, by month, in **A**) PROMISE-1 and **B**) PROMISE-2. MHDs, monthly headache days

Changes from baseline in frequency category over Months 1–6 in PROMISE-1 are illustrated in Fig. 1. At Month 1, 62/221 (28.1%) patients treated with eptinezumab 100 mg and 75/222 (33.8%) patients treated with eptinezumab 300 mg had ≤ 3 MHDs, with 97/221 (43.9%) and 108/222 (48.6%), respectively, falling below this diagnostic EM threshold at Month 6. The proportions of patients in the placebo group achieving this status were numerically lower, at 20.3% (45/222) and 37.8% (84/222) at Months 1 and 6, respectively.

**Fig. 2:**
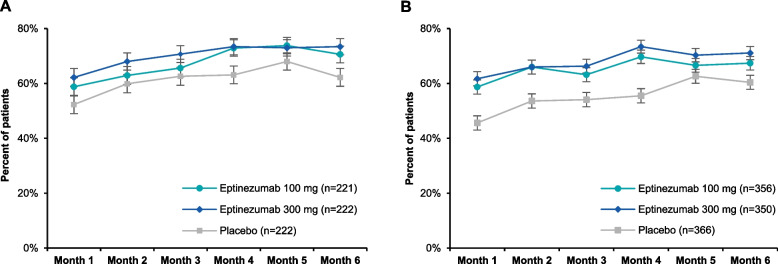
Percentage of patients with reduction of ≥ 1 MHD frequency category in **A**) PROMISE-1 and **B**) PROMISE-2. MHDs, monthly headache days

**Fig. 3:**
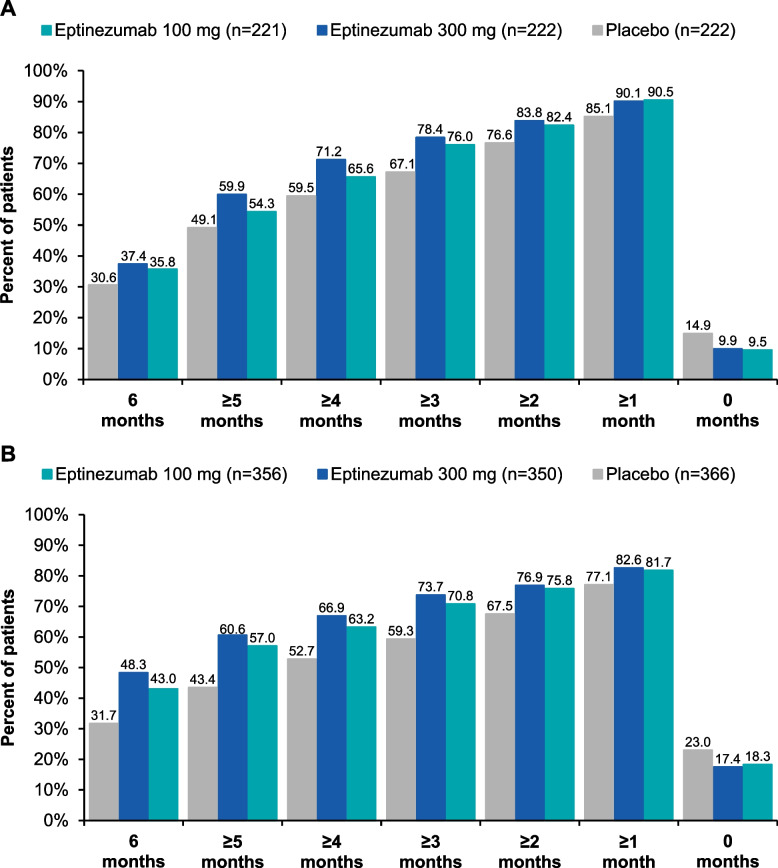
Cumulative number of months with reduction of ≥ 1 MHD frequency category in **A**) PROMISE-1 and **B**) PROMISE-2. MHDs, monthly headache days

A total of 130/221 (58.8%), 138/222 (62.2%), and 116/222 (52.3%) patients in the eptinezumab 100-mg, 300-mg, and placebo groups, respectively, had a reduction of ≥ 1 frequency category at Month 1 and 156/221 (70.6%), 163/222 (73.4%), and 138/222 (62.2%) at Month 6 (Fig. 2). Furthermore, 79/221 (35.7%), 83/222 (37.4%), and 68/222 (30.6%) patients in the eptinezumab 100-mg, 300-mg, and placebo groups, respectively, had 6 months of sustained reduction of ≥ 1 frequency category (Fig. 3).

### Changes in diagnostic category in PROMISE-2

Changes from baseline in frequency category over Months 1–6 in PROMISE-2 are summarized in Fig. 1. At Month 1, 209/356 (58.7%) patients treated with eptinezumab 100 mg and 216/350 (61.7%) treated with eptinezumab 300 mg had ≤ 14 MHDs, with 240/356 (67.5%) and 249/350 (71.1%), respectively, falling below this diagnostic threshold at Month 6. The proportions of patients in the placebo group achieving this status were numerically lower (45.6% [167/366] and 60.4% [221/366]) at Months 1 and 6, respectively. At Month 1, 25.8%, 23.1%, and 23.2% of patients in the eptinezumab 100-mg, 300-mg, and placebo groups met the frequency criteria for HFEM and 23.9%, 26.9%, and 20.5% met the criteria from LFEM, respectively. At Month 6, the shift to LFEM was more pronounced (34.3%, 29.1%, and 25.1%, respectively).

A total of 209/356 (58.7%), 216/350 (61.7%), and 167/366 (45.6%) patients in the eptinezumab 100-mg, 300-mg, and placebo groups, respectively, had a reduction of ≥ 1 frequency category at Month 1 and 240/356 (67.4%), 249/350 (71.1%), and 221/366 (60.4%) at Month 6 (Fig. 2). Furthermore, 153/356 (43.0%), 169/350 (48.3%), and 116/366 (31.7%) patients in the eptinezumab 100-mg, 300-mg, and placebo groups, respectively, had 6 months of reduction of ≥ 1 frequency category (Fig. 3).

**Fig. 4:**
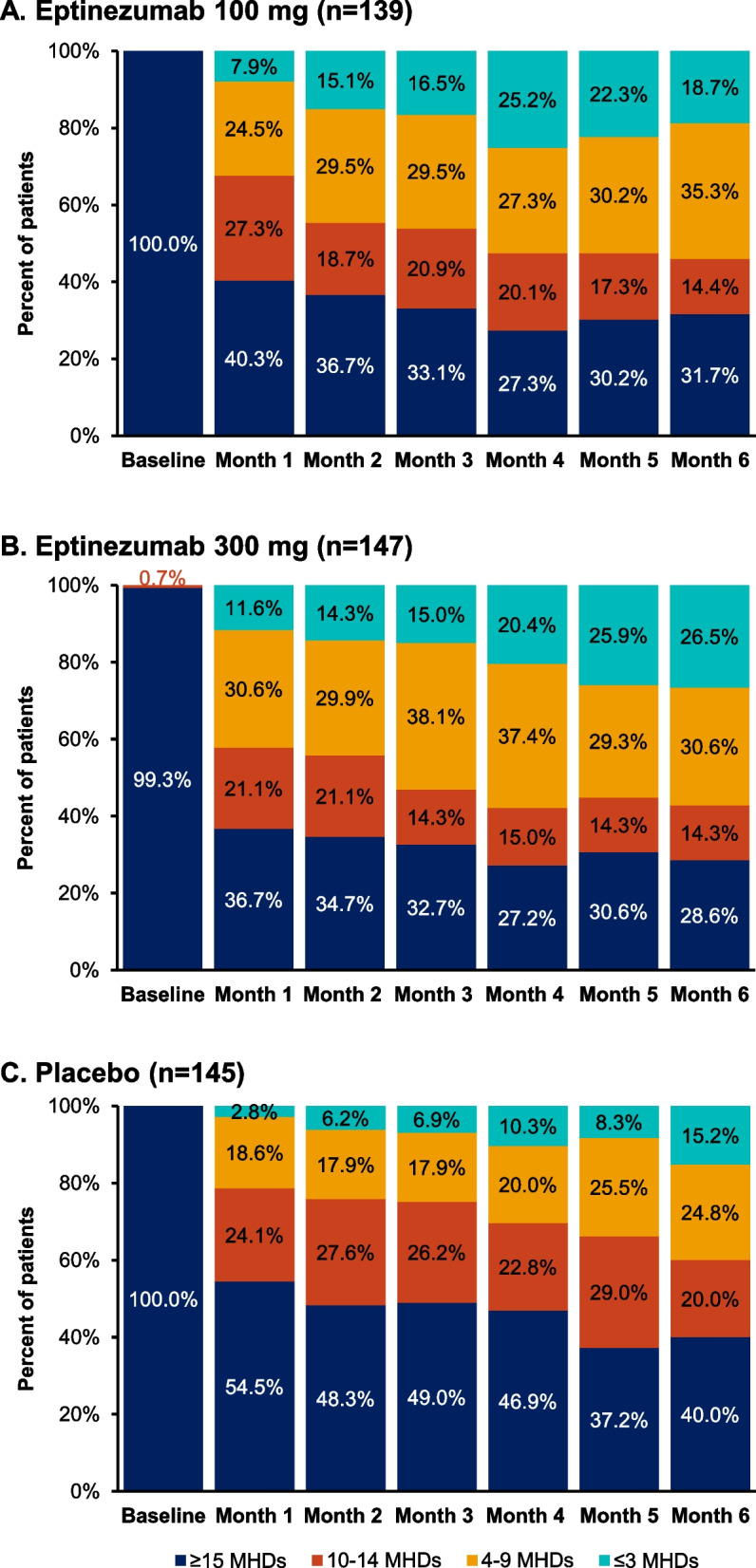
Percentage of patients in each diagnostic category, by month, in the subgroup of patients with MOH diagnosis at baseline in PROMISE-2: **A**) eptinezumab 100 mg; **B**) eptinezumab 300 mg; **C**) placebo. MHDs, monthly headache days; MOH, medication-overuse headache

Changes from baseline in frequency category over Months 1–6 in the subset of patients with MOH in PROMISE-2 are summarized in Fig. 4. At Month 1, 93/139 (63.3%) patients treated with eptinezumab 100 mg and 93/147 (63.3%) patients treated with eptinezumab 300 mg had ≤ 14 MHDs, with 95/139 (68.4%) and 105/147 (71.4%), respectively, falling below this diagnostic threshold at Month 6. The proportions of patients in the placebo group achieving this status were lower, at 45.5% (66/145) and 60.0% (87/145) at Months 1 and 6, respectively.

## Discussion

In this post hoc analysis of data from the PROMISE studies, eptinezumab use was associated with downward shifts in frequency-based classifications that were sustained across two dosing intervals (6 months). The results reported here are in alignment with the responses observed in previous work [26], which showed consistency in percent reduction across subgroups which were defined by baseline headache/migraine frequency. In PROMISE-1, reductions in migraine frequency were evident the first month after eptinezumab initiation and were of sufficient magnitude to render nearly one-third (30.9%) of patients below the threshold of ≤ 4 MHDs often used as the threshold for initiating preventive treatment. Maintenance of this benefit was demonstrated by the similar or greater proportions of patients experiencing ≤ 3 headache days during each subsequent month of the analysis period (36.6%, 38.8%, 46.5%, 49.0%, and 46.3% during Months 2, 3, 4, 5, and 6, respectively). Among those treated with eptinezumab remaining above this threshold, fewer experienced ≥ 10 headache days each month (HFEM or CM) relative to baseline (55.3% at baseline; 19.9%, 18.5%, 18.1%, 13.3%, 14.4%, and 14.9% during Months 1, 2, 3, 4, 5, and 6, respectively).

In patients with CM (PROMISE-2), sustained reductions in headache frequency with eptinezumab were of sufficient magnitude and duration to permit many patients to fall within the range typically considered EM (≤ 14 MHDs) beginning the first month after treatment initiation. Specifically, more than half (60.2%) had ≤ 14 MHDs during the first month after eptinezumab initiation and 66.0%, 64.7%, 71.5%, 68.4%, and 69.3% had ≤ 14 MHDs during Months 2, 3, 4, 5, and 6, respectively. Some CM patients even improved to the point that they fell below the frequency threshold typically used for the indication of preventive treatment (≤ 4 MHDs), with 10.3% achieving this status during Month 1 and 15.3%, 15.3%, 23.4%, 22.5%, and 22.9% during Months 2, 3, 4, 5, and 6, respectively. While these reductions in MHD associated with eptinezumab use likely reduce migraine-related burden, it is important to note that these data should not be interpreted as indicating a patient should discontinue preventive migraine treatment. Based on previous analyses [27], we would also expect some patients to fluctuate between categories despite treatment.

In addition, whereas these findings are suggestive of clinically meaningful changes in headache frequency, they raise some interesting questions for future research. In both studies, marked differences between Months 3 and 4 suggest that the administration of the second dose may not only sustain improvements, but further reduce headache frequency, i.e., an additive effect. These results are supportive of the updated American Headache Society [11] and European Headache Federation [28] guidelines recommending that trials with monoclonal antibodies targeting calcitonin gene-related peptide last for at least 3 to 6 months. Furthermore, observed improvements in the placebo groups of both studies suggest that factors other than eptinezumab administration likely contributed to observed benefits. Lastly, because lower-frequency categories are associated with better quality of life and lower burden/healthcare resource utilization [3–8], examination of the impact of the observed changes on quality of life and healthcare resource consumption are warranted. The latter may be particularly relevant, as access to preventive treatments is often based on diagnostic classification.

### Limitations

Frequency-based classification of migraine is complex, and factors such as severity and associated disability are an important part of defining migraine. Analysis of data from the PROMISE-2 study indicated that reductions in headache frequency were associated with decreases in pain severity; common symptoms such as nausea, phonophobia, and photophobia; and activity limitations [29]. The categories used in this analysis were based on the conceptional framework for transitions in migraine put forth by Bigal and colleagues (2008) [1], with the LFEM category being further broken down to identify patients who fall below the threshold for initiating migraine prevention (≤ 4 MHDs) [11]. To date, no clear definitions of LFEM and HFEM exist, and there is some variability in the range of each category [30–34]. Further, CM subgroups could have been further subdivided into two categories (15–23 MHDs and 24–28 MHDs) which may better capture differences in disease burden, as was described in Ishii et al. 2021 [35]. Although changes in classification based on migraine days were not explored, previous data indicate that MMD reduction parallels MHD reduction and thus would be expected to demonstrate similar improvements. Future work would be needed to determine if sociodemographic or baseline characteristics can be used to predict which patients experience the greatest shifts in diagnostic classification; however, previous work has suggested that such predictors are not easily identified [26, 36].

## Conclusion

Changes in headache frequency during the first 6 months of eptinezumab treatment in the PROMISE studies were frequently of sufficient magnitude and duration to permit a shift in frequency and reclassify to categories associated with better quality of life and reduced healthcare resource utilization.

## Data Availability

Data Sharing Statement: In accordance with EFPIA’s and PhRMA’s “Principles for Responsible Clinical Trial Data Sharing” guidelines, Lundbeck is committed to responsible sharing of clinical trial data in a manner that is consistent with safeguarding the privacy of patients, respecting the integrity of national regulatory systems, and protecting the intellectual property of the sponsor. The protection of intellectual property ensures continued research and innovation in the pharmaceutical industry. Deidentified data are available to those whose request has been reviewed and approved through an application submitted to https://www.lundbeck.com/global/our-science/clinical-data-sharing.

## Abbreviations

CM: Chronic migraine
EM: Episodic migraine
HFEM: High-frequency episodic migraine
LFEM: Low-frequency episodic migraine
MMDs: Monthly migraine days
MOH: Medication-overuse headache
MHD: Monthly headache day

## References

[1] Bigal ME, Lipton RB (2008). Clinical course in migraine: conceptualizing migraine transformation. Neurol.

[2] Serrano D, Lipton RB, Scher AI (2017). Fluctuations in episodic and chronic migraine status over the course of 1 year: implications for diagnosis, treatment and clinical trial design. J Headache Pain.

[3] Buse DC, Reed ML, Fanning KM (2020). Demographics, headache features, and comorbidity profiles in relation to headache frequency in people with migraine: results of the American Migraine Prevalence and Prevention (AMPP) study. Headache.

[4] Buse DC, Fanning KM, Reed ML (2019). Life with migraine: effects on relationships, career, and finances from the chronic migraine epidemiology and outcomes (CaMEO) study. Headache.

[5] Buse DC, Reed ML, Fanning KM (2020). Comorbid and co-occurring conditions in migraine and associated risk of increasing headache pain intensity and headache frequency: results of the migraine in America symptoms and treatment (MAST) study. J Headache Pain.

[6] Torres-Ferrús M, Quintana M, Fernandez-Morales J (2017). When does chronic migraine strike? A clinical comparison of migraine according to the headache days suffered per month. Cephalalgia.

[7] Blumenfeld AM, Varon SF, Wilcox TK (2011). Disability, HRQoL and resource use among chronic and episodic migraineurs: Results from the International Burden of Migraine Study (IBMS). Cephalalgia.

[8] Silberstein SD, Lee L, Gandhi K (2018). Health care resource utilization and migraine disability along the migraine continuum among patients treated for migraine. Headache.

[9] National Headache Foundation Position Statement on the Treatment of Migraine and Access to Care. National Headache Foundation; 2022. https://headaches.org/national-headache-foundation-position-statement-on-the-treatment-of-migraine/. Accessed 28 Feb 2022.

[10] Pringsheim T, Davenport W, Mackie G (2012). Canadian headache society guideline for migraine prophylaxis. Can J Neurol Sci.

[11] Ailani J, Burch RC, Robbins MS (2021). The American headache society consensus statement: update on integrating new migraine treatments into clinical practice. Headache.

[12] Silberstein SD (2015). Preventive migraine treatment. Continuum (Minneap Minn).

[13] Vyepti [package insert]. Lundbeck Seattle BioPharmaceuticals Inc; 2021.

[14] Vyepti [EMA Authorization]. Lundbeck A/S Valby, Denmark; 2021.

[15] Product Monograph Including Patient Medication Information: Vyepti (Eptinezumab for injection). Lundbeck Canada Inc; 2021.

[16] Dodick DW, Lipton RB, Silberstein S (2019). Eptinezumab for prevention of chronic migraine: a randomized phase 2b clinical trial. Cephalalgia.

[17] Lipton RB, Goadsby PJ, Smith J (2020). Efficacy and safety of eptinezumab in patients with chronic migraine: PROMISE-2. Neurol.

[18] Ashina M, Saper J, Cady R (2020). Eptinezumab in episodic migraine: a randomized, double-blind, placebo-controlled study (PROMISE-1). Cephalalgia.

[19] Smith TR, Janelidze M, Chakhava G (2020). Eptinezumab for the prevention of episodic migraine: sustained effect through 1 year of treatment in the PROMISE-1 study. Clin Ther.

[20] Silberstein S, Diamond M, Hindiyeh NA (2020). Eptinezumab for the prevention of chronic migraine: efficacy and safety through 24 weeks of treatment in the phase 3 PROMISE-2 (Prevention of migraine via intravenous ALD403 safety and efficacy–2) study. J Headache Pain.

[21] Kudrow D, Cady RK, Allan B (2021). Long-term safety and tolerability of eptinezumab in patients with chronic migraine: a 2-year, open-label, phase 3 trial. BMC Neurol.

[22] Winner PK, McAllister P, Chakhava G (2021). Effects of intravenous eptinezumab vs placebo on headache pain and most bothersome symptom when initiated during a migraine attack: a randomized clinical trial. JAMA.

[23] Smith TR, Spierings ELH, Cady R (2021). Safety and tolerability of eptinezumab in patients with migraine: a pooled analysis of 5 clinical trials. J Headache Pain.

[24] Dodick DW, Gottschalk C, Cady R (2020). Eptinezumab demonstrated efficacy in sustained prevention of episodic and chronic migraine beginning on Day 1 after dosing. Headache.

[25] Irimia P, Garrido-Cumbrera M, Santos-Lasaosa S (2021). Impact of monthly headache days on anxiety, depression and disability in migraine patients: results from the Spanish Atlas. Sci Rep.

[26] Martin V, Nagy AJ, Janelidze M (2022). Impact of baseline characteristics on the efficacy and safety of Eptinezumab in patients with migraine: subgroup analyses of PROMISE-1 and PROMISE-2. Clin Ther.

[27] Buse DC, Winner PK, Charleston L (2022). Early response to eptinezumab indicates high likelihood of continued response in patients with chronic migraine. J Headache Pain.

[28] Sacco S, Amin FM, Ashina M (2022). European Headache Federation guideline on the use of monoclonal antibodies targeting the calcitonin gene related peptide pathway for migraine prevention – 2022 update. J Headache Pain.

[29] McAllister P, Kudrow D, Cady R (2022). Reduction in migraine-associated burden after eptinezumab treatment in patients with chronic migraine. Cephalalgia.

[30] Doane MJ, Gupta S, Fang J (2020). The humanistic and economic burden of migraine in Europe: a cross-sectional survey in five countries. Neurol Ther.

[31] Lipton RB, Serrano D, Pavlovic JM (2014). Improving the classification of migraine subtypes: an empirical approach based on factor mixture models in the american migraine prevalence and prevention (AMPP) study. Headache.

[32] Serrano D, Buse DC, Kori SH (2013). Effects of switching acute treatment on disability in migraine patients using triptans. Headache: J Head Face Pain.

[33] Caronna E, Gallardo VJ, Alpuente A (2022). Epidemiology, work and economic impact of migraine in a large hospital cohort: time to raise awareness and promote sustainability. J Neurol.

[34] Katsarava Z, Manack A, Yoon M-S (2011). Chronic migraine: classification and comparisons. Cephalalgia.

[35] Ishii R, Schwedt TJ, Dumkrieger G (2021). Chronic versus episodic migraine: The 15‐day threshold does not adequately reflect substantial differences in disability across the full spectrum of headache frequency. Headache: J Head and Face Pain.

[36] Apelian R, Boyle L, Hirman J, Asher D (2022). Measuring dose-related efficacy of eptinezumab for migraine prevention: post hoc analysis of PROMISE-1 and PROMISE-2. J Headache Pain.

